# Medulloblastoma in children and adolescents: a systematic review of contemporary phase I and II clinical trials and biology update

**DOI:** 10.1002/cam4.1171

**Published:** 2017-10-04

**Authors:** Francisco Bautista, Victoria Fioravantti, Teresa de Rojas, Fernando Carceller, Luis Madero, Alvaro Lassaletta, Lucas Moreno

**Affiliations:** ^1^ CNIO‐HNJ Clinical Research Unit Pediatric Oncology, Hematology and Stem Cell Transplant Department Hospital Infantil Universitario Niño Jesús Avenida Menéndez Pelayo, 65 28009 Madrid Spain; ^2^ Pediatric and Adolescent Drug Development, Children and Young People's Unit The Royal Marsden NHS Foundation Trust London UK; ^3^ Division of Clinical Studies and Cancer Therapeutics The Institute of Cancer Research London UK; ^4^ Instituto de Investigación La Princesa Madrid Spain

**Keywords:** Children, clinical trial, medulloblastoma, phase 1, phase 2, relapse or refractory tumor

## Abstract

Survival rates for patients with medulloblastoma have improved in the last decades but for those who relapse outcome is dismal and new approaches are needed. Emerging drugs have been tested in the last two decades within the context of phase I/II trials. In parallel, advances in genetic profiling have permitted to identify key molecular alterations for which new strategies are being developed. We performed a systematic review focused on the design and outcome of early‐phase trials evaluating new agents in patients with relapsed medulloblastoma. PubMed, clinicaltrials.gov, and references from selected studies were screened to identify phase I/II studies with reported results between 2000 and 2015 including patients with medulloblastoma aged <18 years. A total of 718 studies were reviewed and 78 satisfied eligibility criteria. Of those, 69% were phase I; 31% phase II. Half evaluated conventional chemotherapeutics and 35% targeted agents. Overall, 662 patients with medulloblastoma/primitive neuroectodermal tumors were included. The study designs and the response assessments were heterogeneous, limiting the comparisons among trials and the correct identification of active drugs. Median (range) objective response rate (ORR) for patients with medulloblastoma in phase I/II studies was 0% (0–100) and 6.5% (0–50), respectively. Temozolomide containing regimens had a median ORR of 16.5% (0–100). Smoothened inhibitors trials had a median ORR of 8% (3–8). Novel drugs have shown limited activity against relapsed medulloblastoma. Temozolomide might serve as backbone for new combinations. Novel and more homogenous trial designs might facilitate the development of new drugs.

## Introduction

Medulloblastomas are aggressive embryonal tumors representing the most frequent primary malignant brain cancer in children 1. Maximal safe resection, chemotherapy, and craniospinal irradiation (CSI) remain the mainstays of first‐line treatment 2.

Long‐term survival rates have steadily improved over the last decades, from 22% by 1950 3 to up to 50% by late 1970 4 and even 85% with current approaches 5; this improvement is mostly due to the addition of systemic chemotherapy to the standard treatment with surgery and radiotherapy 6, 7, 8, superior surgical and radiotherapy techniques, intensification of therapy 9, 10, and improvement in supportive care measures. Unfortunately outcome is invariably poor for those who relapse 11, 12, with a long‐term survival of 6% 11 and new approaches are needed .

Clinical trials are the way forward to evaluate new therapies for high‐risk cancer patients 13. Patients with relapsed or refractory brain tumors represent between 36% 14 and 46% 15 of the population participating in pediatric oncology phase I studies; of those, medulloblastoma/primitive neuroectodermal tumors (PNET) patients represent up to a third. Moreover, patients with medulloblastoma and PNET have been traditionally treated together in trials although they are distinct molecular entities and PNETs are now called central nervous system (CNS) embryonal tumors 16.

The advent of the molecular classification 17 and the advances in genetic profiling of medulloblastomas open the horizon for more tailored therapeutic approaches. In this sense, classical criteria used to stratify patients based on residual tumor burden after surgery 18, age, and extent of disease may not accurately identify patients with better or worse outcome. The implementation of molecular variables into stratification schemes can help to refine risk definition and subsequent treatment 19. The identification of good‐prognosis patients may allow de‐escalating the intensity of frontline therapies and reducing long‐term sequelae. Conversely, high‐risk patients may benefit from adding new agents to conventional chemotherapeutics or even substituting those associated with more undesirable side effects by others with a better safety profile, while keeping their antitumor activity.

Hence, the number of potential patients with medulloblastoma for entering early‐phase trials or new therapies targeting a vast landscape of molecular alterations makes necessary an analysis of the activity that has already been carried out.

We performed a systematic review of the methodology and results of phase I/II clinical trials including pediatric patients with medulloblastoma at relapse/progression and we reviewed current molecularly driven trials in this population.

The objectives were as follows:

1. To stablish the level of activity and outcome of phase I/II studies for patients with medulloblastoma in the last 15 years;
2. To provide an update on the medulloblastoma clinical trials portfolio and to discuss current knowledge on biology and potential future targeted therapies;
3. To inform future trials and to discuss potential areas of improvement to optimize early clinical trials performance.

## Material and Methods

### Search strategy

PubMed (https://www.ncbi.nlm.nih.gov/pubmed) was searched with three different strategies to cover medulloblastoma‐specific trials, CNS tumor trials, and solid tumor trials (Data S1). Search was limited to articles published with patients aged <18 years old, between 2000 and 2015. No language restrictions were applied. The https://clinicaltrials.gov site was also searched, restricted to interventional phase I/II studies with results in children with medulloblastoma from 1st January 2000 to 31st December 2015, as well as the bibliographic references from the studies finally included in this review.

One reviewer (VF) evaluated the titles and abstracts of the identified publications and all potential relevant publications were retrieved for detailed evaluation. The final inclusion of studies was made by agreement of two reviewers (VF and FB). A third author (LM) reviewed ‘Potentially relevant publications retrieved for detailed evaluation’ independently and blindly to peer review the inclusion of papers. Two reviewers performed the data abstraction (VF and FB) by means of a standardized data collection form.

### Inclusion and exclusion criteria

Inclusion and exclusion criteria were defined a priori. Phase I/II trials including patients with medulloblastoma aged <18 years at the time of enrolment were eligible. Stand‐alone radiotherapy trials were excluded.

### Data extraction

Information was extracted regarding study design, inclusion/exclusion criteria, target population, type of intervention, outcome, and toxicity. Objective response rate (ORR) was calculated as the proportion of complete responses (CR) and partial responses (PR) among evaluable patients. Disease control rate (DCR) was calculated as the proportion of CR, PR, and stable diseases (SD) among evaluable patients.

### Review of current molecularly driven trials in patients with medulloblastoma

The website https://clinicaltrials.gov was scrutinized to identify ongoing trials, using the advance search function. We used the term “medulloblastoma” and restricted our search to studies that were not yet recruiting or recruiting limited to the age group of child (birth–17 years); last accessed on 28th July 2017.

## Results

### Included studies

**Figure 1:**
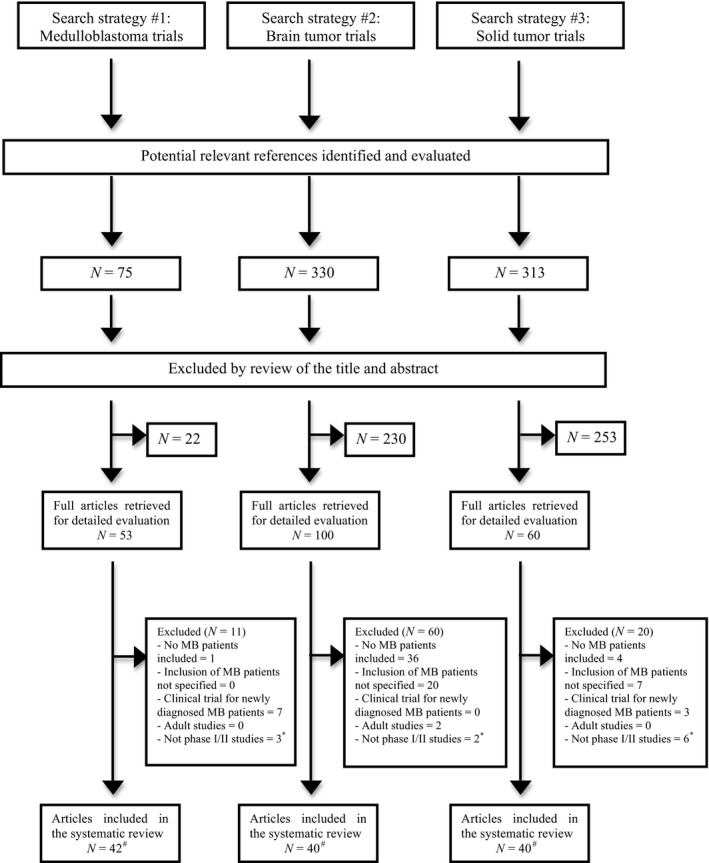
Flow diagram reporting results of the systematic review. MB, medulloblastoma. *In this category felt retrospective or observational studies. ^#^Some studies finally included in the systematic review were identified by one or more search strategies. Therefore, there is an overlap of identified studies among research strategies yielding a final number of individual studies of 78.

A total of 718 publications were identified (Data S1). Two hundred and thirteen articles were retrieved for detailed evaluation; 78 satisfied eligibility criteria. Adapted PRISMA flow diagram displays the process (Fig. 1) for including studies 20. Nine studies with results were identified in https://clinicaltrials.gov. Five had already been identified in Pubmed 21, 22, 23, 24, 25, 26 and one other (NCT01125800) had also been presented elsewhere 27. In three studies the data about patients with medulloblastoma were not available and they could not be analyzed (NCT01483820, NCT00867568, and NCT00024258).

### Clinical trials description

There were 54 phase I (69%) 21, 26, 27, 28, 29, 30, 31, 32, 33, 34, 35, 36, 37, 38, 39, 40, 41, 42, 43, 44, 45, 46, 47, 48, 49, 50, 51, 52, 53, 54, 55, 56, 57, 58, 59, 60, 61, 62, 63, 64, 65, 66, 67, 68, 69, 70, 71, 72, 73, 74, 75, 76, 77, 78 and 24 phase II clinical trials (31%) 22, 23, 24, 25, 79, 80, 81, 82, 83, 84, 85, 86, 87, 88, 89, 90, 91, 92, 93, 94, 95, 96, 97, 98. Half evaluated conventional chemotherapeutics (*n* = 40) and 35% (*n* = 27) targeted therapies (Table 1).

**Table 1:** Clinical trials baseline characteristics and patient population description

Description of clinical trials included in this study			Patient population description			
			All patients		Medulloblastoma patients^a^	
Variable	*N*	%	*N*	%	*N*	%
Total studies included in the analysis	78	100	3531	100	662	100
Participating centers						
Unicenter	9	12	148	4	30	5
Multicenter	69	88	3383	96	632	95
Phase of development						
Phase I	54	69	1714	48	261	39
Phase II	24	31	1817	52	401	61
Randomization						
Yes	1	1	44	1	12	2
No	77	99	3487	99	650	98
Age at inclusion						
Up to 18 years	10	12	380	10	139	21
Up to 21 years	59	76	2906	83	464	70
>22 years	9	12	245	7	59	9
Target population categories						
Medulloblastoma	4	5	125	4	125	19
Central Nervous System tumors	33	43	1452	41	325	49
Solid tumors (CNS and extra‐CNS)	41	52	1954	56	212	32
Class of therapeutic(s) agent(s)						
Conventional chemotherapeutic single agent	24	31	1510	43	277	42
Conventional chemotherapeutics combination	15	19	631	18	134	20
Targeted agent monotherapy	25	32	880	25	164	25
Targeted agents in combination	2	3	29	1^#^	2	0^#^
Chemotherapeutics + targeted agent in combination	9	11	401	11	36	5
Chemotherapeutics + HSCT	3	4	80	2	49	7

a 96 out of the 662 patients included were presented in the original manuscript as medulloblastoma/PNET and figures could not be split.

Relative value expressed in percentage is 0.8%. # Relative value expressed in percentage is 0.3

### Clinical trials design

The majority of phase I dose‐escalation trials followed a 3 + 3 design (*n* = 32, 60%), continual reassessment method (*n* = 9, 17%), or rolling six design (*n* = 8, 15%).

The majority of phase II studies followed a two‐stage Simon optimal design (*n* = 20, 83%). In four studies (6%) the design was not specified. The true response rate to declare the drug active ranged between 20% and 40% with probabilities ranging from 80% to 95%. Response was assessed by RECIST criteria (*n* = 5, 21%), World Health Organization (WHO) guidelines (*n* = 18, 75%), or other (*n* = 1, 4%) (Tables 2 and 3).

**Table 2:** Intervention, population, design, and baseline characteristics of phase I studies including patients with medulloblastoma

Drug(s)	Population & design		Baseline characteristics (All patients)					Patients with medulloblastoma		Reference (Year of publication)
	Disease type	Statistical design	*N*	Median age (Y)	Range	Male/Female	Median prior Tx	*N*	% among all patients	
Conventional chemotherapeutic single agent										
Temozolomide	CNS	3 + 3	27	10.8	4–19	13/14	1	6	22	28 (2006)
Fotemustine	CNS	3 + 3	16	5	1.8–14.5	6/9	NA	6	38	29 (2009)
Cloretazine	CNS	CRM	42	9.9	1.5–21.5	20/22	NA	7	16	30 (2008)
Irinotecan	All Tm	3 + 3	81	7.9	0.9–18.5	50/31	2	19	23	31 (2003)
Liposomal Daunorubicine	All Tm	3 + 3	48	9.6	1.3–18.5	28/20	NA	2	4	32 (2006)
Plitidepsin	All Tm	3 + 3	41	10	2–17	21/20	3	3	7	33 (2012)
Depsipeptide	All Tm	3 + 3	24	13	2–21	11/12	NA	1	4	34 (2006)
Fenretidine	All Tm	3 + 3	54	9	2–20	35/19	NA	2	3	35 (2006)
Pemetrexed	All Tm	3 + 3	33	12	1–21	21/12	2	1	3	36 (2007)
Oxaliplatin	All Tm	3 + 3	26	11	5–21	17/9	NA	5	19	37 (2007)
Satraplatin	All Tm	3 + 3	9	17	8–19	5/4	2	1	11	38 (2015)
Intrathecal lyposomal Ara‐C	All Tm	3 + 3	18	10	4–19	12/6	NA	7	39	39 (2004)
Conventional chemotherapeutics combination										
TMZ + VP‐16	MB	3 + 3	14	7.3	3–16.1	8/6	NA	14	100	40 (2010)
O6‐Benzylguanine + TMZ	CNS	CRM	70	11.3	2.4–18.6	43/27	NA	10	14	41 (2007)
Cisplatin + Topotecan	All Tm	3 + 3	36	12	2–21	20/16	NA	1	3	42 (2002)
Irinotecan + Cisplatin	All Tm	3 + 3	24	15	4–21	10/14	NA	1	4	43 (2003)
CPM + Topotecan	All Tm	3 + 3	16	11.9	2.8–18	10/6	2	3^a^	2	44 (2004)
Cisplatin + TMZ	All Tm	CRM	39	12.7	1.8–19.9	25/14	NA	2	5	45 (2005)
Carboplatin + Irinotecan	All Tm	3 + 3	28	8.5	1–21	17/11	NA	2	7	46 (2009)
Oxaliplatin + VP16	All Tm	3 + 3	16	8	1–18	11/5	3	3	19	47 (2009)
Oxaliplatin + Irinotecan	All Tm	3 + 3	13	16	5–21	4/9	1	1	8	48 (2009)
Irinotecan + TMZ + VCR	All Tm	3 + 3	42	9.7	1–21	23/19	2	2	5	49 (2010)
Oxaliplatin + Ifosfamide + VP16	All Tm	3 + 3	17	7	2–21	12/5	3	2	12	50 (2015)
Targeted agent monotherapy										
Vismodegib	MB	NA	33	13	4.4–20.3	25/8	NA	33	100	26 (2013)
Lonarfarnib	CNS	CRM	53	12.2	3.9–19.5	32/21	NA	2	4	51 (2007)
Cilengitide	CNS	CRM	33	7.9	0.2–21.2	22/11	NA	3	9	52 (2008)
Lapatinib	CNS	CRM	59	9.5	1.1–21.2	30/29	NA	15^a^	25	21 (2010)
Valproic acid	CNS	R‐six	26	13.5	3–21	10/16	3	2	8	53 (2011)
MK‐0752	CNS	CRM	23	8.1	2.6–17.7	10/13	NA	4^a^	17	54 (2011)
MK‐0752	CNS	R‐six	10	8.8	3.1–19.2	6/4	2	1	10	78 (2015)
Erlotinib	CNS	3 + 3	29	10	4–20	15/14	1	1	3	55 (2011)
Lenalidomide	CNS	CRM	51	10.4	2.7–21.6	26/25	3	6^a^	11	56 (2011)
Pazopanib	CNS	R‐six	51	12.9	3.8–23.9	26/25	2	1	2	57 (2013)
Enzastaurin	CNS	CRM	33	12	3–21	16/17	NA	1	3	58 (2015)
PTC299	CNS	R‐six	27	11.2	5.5–21.1	14/13	2	1	4	59 (2015)
Dendritic cells	CNS	NA	9	15.5	9–22	1/8	NA	1	11	60 (2004)
3F8 monoclonal antibody	CNS	NA	15	NA	1–61	NA	NA	4	27	61 (2007)
RG1507	All Tm	3 + 3	31	11	3–17	17/14	NA	1	3	62 (2011)
AT9283	All Tm	R‐six	33	9	3–18	11/22	4	2	6	63 (2015)
Sonidegib	All Tm	Bayesian	33	13	4–17	NA	NA	24	73	27 (2010)
SU101	All Tm	3 + 3	27	14	3–21	19/8	3	4	15	64 (2004)
Temsirolimus	All Tm	3 + 3	19	11	4–21	11/8	NA	2	11	65 (2011)
MK‐2206	All Tm	R‐six	50	14.3	3.1–21.9	26/24	NA	3^a^	6	66 (2014)
Vorinostat ± retinoic acid	All Tm	3 + 3	63	11	2.6–22	40/23	2	9	14	67 (2010)
Targeted agent combination										
Temsirolimus + Bevacizumab	CNS	NA	6	6	3–14	NA	NA	2	33	68 (2014)
Vorinostat + Bortezomib	All Tm	R‐six	23	12.6	1.1–20.1	17/6	NA	1	4	77 (2013)
Chemotherapeutics + targeted agent in combination										
Vorinostat + TMZ	CNS	R‐six	19	8.3	2.1–20.8	12/7	1	2	11	69 (2013)
Veliparib + TMZ	CNS	3 + 3	31	8.5	1.8–21	16/15	1	2	6	70 (2014)
Carboplatin + Thalidomide	All Tm	3 + 3	22	11	5–17	13/9	2	4	18	71 (2004)
Erlotinib + TMZ	All Tm	3 + 3	46	11.5	3–20	30/16	NA	6	13	72 (2008)
VIT + Bevacizumab	All Tm	3 + 3	12	11	3.9–19.4	8/4	2	1	8	73 (2013)
Bevacizumab + Irinotecan	All Tm	3 + 3	11	9	3–22	5/6	NA	2	18	74 (2013)
Temsirolimus + Irinotecan + TMZ	All Tm	3 + 3	71	11	1–21.5	45/26	2	2	3	75 (2014)
Chemotherapeutics + HSCT										
Thiotepa + Carmustine + Carboplatin	CNS	3 + 3	32	7	1.75–18	16/16	NA	18	56	76 (2011)

All Tm, all tumors; CPM, cyclophosphamide; CRM, continual reassessment method; HSCT, hematopoietic stem cell transplantation; MB, medulloblastoma; NA, not available; R‐six, rolling six method; TMZ, temozolomide; Tx, therapies; VCR, vincristine; VIT, Vincristine + Temozolomide + Irinotecan; Y, years.

a Medulloblastoma/PNET cohort that could not be split with the data obtained from the report.

**Table 3:** Intervention, population, design, and baseline characteristics of phase II studies including patients with medulloblastoma

Drug(s)	Population & design		Baseline characteristics (All patients)					Patients with medulloblastoma		Reference (Year of publication)
	Disease type	True response rate to declare the drug active (%) (Probability,%)	*N*	Median age (Y)	Range	Male/Female	Median prior Tx	*N*	% among all patients	
Conventional chemotherapeutic single agent										
Oral methotrexate	CNS	30 (90)	82	NA	NA	NA	NA	18	22	79 (2000)
Placitaxel	CNS	30 (95)	73	7.7	0.3–19	41/32	NA	16	22	80 (2001)
Idarubicin	CNS	30 (87)	91	NA	3–19	50/41	NA	21	23	81 (2003)
Oxaliplatin	CNS	35 (95)	43	8.5	0.6–18.9	30/13	NA	30	70	82 (2006)
Temozolomide	CNS	30 (95)	121	11	1–23	63/85	NA	29^a^	24	83 (2007)
Temozolomide	CNS	25 (80)	40	10	2–21	31/9	NA	37	93	84 (2014)
Topotecan	All Tm	30 (95)	53	12.9	2–23	23/30	NA	2	4	85 (2006)
Docetaxel	All Tm	30 (95)	173	13	1–27	107/66	NA	20	12	86 (2006)
Irinotecan	All Tm	30 (80)	161	9	1–23	104/67	NA	25^a^	16	87 (2007)
Rebeccamycin analog	All Tm	25 (88)	133	9	0–21	72/61	NA	7	5	88 (2008)
Vinorelbine	All Tm	30 (88)	50	8.5	0–20	24/26	NA	2	4	89 (2009)
Pemetrexed	All Tm	30 (95)	72	11	3–23	39/33	NA	10	14	23 (2013)
Conventional chemotherapeutics combination										
Temozolomide + Irinotecan	MB	30 (80)	66	10.5	2–17	45/21	NA	66	100	24 (2013)
Lobradimil + Carboplatin	CNS	20 (90)	40	9	2–21	20/20	NA	6^a^	15	90 (2006)
Gemcitabine + Oxaliplatin	All Tm	40 (95)	93	11.7	1.3–20.8	52/41	NA	14	15	91 (2011)
Vinorelbine + CPM	All Tm	NA	117	12	1–24	61/56	NA	7	6	92 (2012)
Targeted agent monotherapy										
Tipifarnib	CNS	25 (95)	97	11.2	3.2–21.9	45/52	NA	12	12	93 (2007)
Imatinib	CNS	NA	19	9	2–18	12/7	2	8^a^	42	94 (2009)
Lapatinib	CNS	25 (90)	44	9.4	1.2–21.3	20/24	NA	12	27	22 (2013)
Vismodegib	MB	25 (90)	12	10.4	3.9–20	6/6	NA	12	100	98 (2015)
Targeted agent combination (*n* = 0)										
Chemotherapeutics + targeted agent in combination										
Bevacizumab + Irinotecan	CNS	NA	92	NA	0.6–20.1	NA	NA	10	11	25 (2013)
Multiagent metronomic	All Tm	30 (95)	97	10	0–21	50/47	NA	6	6	95 (2014)
Chemotherapeutics + HSCT										
Multiagent conditioning	CNS	NA	19	NA	0.2–17	13/6	NA	9	47	96 (2010)
CPM + Melphalan	CNS	NA	29	9.8	4.3–17.1	17/12	NA	22	76	97 (2008)

All Tm, all tumors; CPM, cyclophosphamide; HSCT, hematopoietic stem cell transplantation; MB, medulloblastoma; NA, not available; OR, objective response; Tx, therapies; Y, years.

a Medulloblastoma/PNET cohort that could not be split with the data obtained from the report.

### Study population

A total of 3531 patients were included in the 78 studies that satisfied the eligibility criteria. Of those, 566 patients (16%) had medulloblastoma. In nine studies (12%), medulloblastoma and CNS‐PNET patients (*n* = 96) were presented together and figures could not be split; all were included in the analysis (Total = 662 patients). The proportion of patients with medulloblastoma was 11% in trials for patients with solid tumors (*n* = 212/1954 patients) and 22% in CNS tumors trials (*n* = 325/1452 patients). Median number of patients with medulloblastoma per trial was 4 (range, 1–66).

### Response and outcome in patients with medulloblastoma

Data about response could be extracted from 48 of 54 phase I studies (89%) and 21 of 24 phase II (88%) (Tables 4 and 5). Median ORR (range) for all patients with medulloblastoma (*n* = 662) was 0% (0–100). Median ORR (range) in phase I studies was 0% (0–100) and 6.5% (0–50) in phase II. Median DCR in phase I studies was 16% (0–100) and 25% (0–75) in phase II.

**Table 4:** Response rates of phase I studies including patients with medulloblastoma

	*N* (MB patients)	CR	PR	SD	PD	Objective response rate (%)	Disease control rate (%)	Reference (year of publication)
Conventional chemotherapeutic single agent								
Temozolomide	6	2	0	NA	NA	33	33	28 (2006)
Fotemustine	6	0	0	1	5	0	16	29 (2009)
Cloretazine	7	0	0	1	6	0	14	30 (2008)
Irinotecan	19	0	1	1	17	5	11	31 (2003)
Liposomal Daunorubicine	2	NA	NA	NA	NA	NA	NA	32 (2006)
Plitidepsin	3	0	0	1	2	0	33	33 (2012)
Depsipeptide	1	0	0	0	1	0	0	34 (2006)
Fenretidine	2	0	0	0	2	0	0	35 (2006)
Pemetrexed	1	0	0	0	1	0	0	36 (2007)
Oxaliplatin	5	0	0	1	4	0	20	37 (2007)
Satraplatin	1	0	0	1	0	0	100	38 (2015)
Intrathecal lyposomal Ara‐C	7	0	0	2	5	0	29	39 (2004)
Total	60	2	1	8	43	–	–	
ORR/DCR^b^	–	ORR(3/58 = 5%		DCR 11/58 = 19%	–	–	–	
Median objective response/disease control rate (Range)^c^						0 (0–33)	16 (0–100)	
Conventional chemotherapeutics combination								
TMZ + VP16	14	1	1	7	3	17^a^	75	40 (2010)
O6‐Benzylguanine + TMZ	10	0	0	2	8	0	20	41 (2007)
Cisplatin + Topotecan	1	0	0	0	1	0	0	42 (2002)
Irinotecan + Cisplatin	1	0	0	1	0	0	100	43 (2003)
CPM + Topotecan	3^d^	0	0	1	2	0	33	44 (2004)
Cisplatin + TMZ	2	0	0	0	2	0	0	45 (2005)
Carboplatin + Irinotecan	2	1	1	0	0	100	100	46 (2009)
Oxaliplatin + VP16	3	1	0	0	2	33	33	47 (2009)
Oxaliplatin + Irinotecan	1	0	0	0	1	0	0	48 (2009)
Irinotecan + TMZ + VCR	2	0	0	2	0	0	100	49 (2010)
Oxaliplatin + Ifosfamide + VP16	2	0	1	0	1	50	50	50 (2015)
Total	41	3	3	13	20	–	–	
ORR/DCR	–	ORR 6/39 = 15%		DCR 19/39 = 48%	–	–	–	
Median objective response/disease control rate (Range)^c^						0 (0–100)	33 (0–100)	
Targeted agent monotherapy								
Vismodegib	33	1	0	0	32	3	3	26 (2013)
Lonarfarnib	2	0	0	1	1	0	50	51 (2007)
Cilengitide	3	0	0	1	2	0	33	52 (2008)
Lapatinib	15^d^	0	0	1	14	0	7	21 (2010)
Valproic acid	2	0	0	0	2	0	0	53 (2011)
MK‐0752	4^d^	0	0	0	4	0	0	54 (2011)
Erlotinib	1	NA	NA	NA	NA	NA	NA	78 (2015)
Lenalidomide	6^d^	NA	NA	NA	NA	NA	NA	55 (2011)
Pazopanib	1	0	0	0	1	0	0	56 (2011)
Enzastaurin	1	0	0	0	1	0	0	57 (2013)
PTC299	1	0	0	0	1	0	0	58 (2015)
Dendritic cells	1	NA	NA	NA	NA	NA	NA	59 (2015)
3F8 monoclonal antibody	4	0	0	0	4	0	0	60 (2004)
MK‐0752	1	0	0	0	1	0	0	61 (2007)
RG1507	1	NA	NA	NA	NA	NA	NA	62 (2011)
AT9283	2	0	0	0	2	0	0	63 (2015)
Sonidegib	24	2	0	0	22	8	8	27 (2010)
SU101	4	0	0	1	3	0	25	64 (2004)
Temsirolimus	2	0	0	NA	NA	0	NA	65 (2011)
MK‐2206	3^d^	0	0	0	3	0	0	66 (2014)
Vorinostat ± retinoic acid	9	0	0	1	8	0	11	67 (2010)
Total	120	3	0	5	101	–	–	
ORR/DCR	–	ORR 3/110 = 2.8%		DCR)8/110 = 7%	–	–	–	
Median objective response/disease control rate (Range)^c^						0 (0–8)	0 (0–50)	
Targeted agent combination								
Temsirolimus + Bevacizumab	2	0	0	1	1	0	50	68 (2014)
Vorinostat + Bortezomib	1	0	0	0	1	0	0	77 (2013)
Total	2	0	0	1	1	–	–	
ORR/DCR	–	ORR 0/3 = 0%		DCR 1/3 = 33%	–	–	–	
Median objective response/disease control rate (Range)^c^						0 (0–0)	25 (0–50)	
Chemotherapeutics + targeted agent in combination								
Vorinostat + TMZ	2	0	0	0	2	0	0	69 (2013)
Veliparib + TMZ	2	NA	NA	NA	NA	NA	NA	70 (2014)
Carboplatin + Thalidomide	4	0	0	1	3	0	25	71 (2004)
Erlotinib + TMZ	6	0	1	0	5	17	17	72 (2008)
VIT + Bevacizumab	1	0	1	0	0	100	100	73 (2013)
Bevacizumab + Irinotecan	2	0	0	1	1	0	50	74 (2013)
Temsirolimus + Irinotecan + TMZ	2	0	0	0	2	0	0	75 (2014)
Total	19	0	2	2	13	–	–	
ORR/DCR	–	ORR 2/17 = 12%		DCR 4/17 = 24%	–	–	–	
Median objective response/disease control rate (Range)^c^						0 (0–100)	21 (0–100)	
Chemotherapeutics + HSCT								
Thiotepa + Carmustine + Carboplatin	18	4	0	0	14	22	22	76 (2011)
Total	18	4	0	0	14	–	–	
ORR/DCR	–	ORR 4/18 = 22%		DCR 4/18 = 22%				
Median objective response/disease control rate (Range)^c^						22	22	

CPM, cyclophosphamide; CR, complete response; DCR, disease control rate; HSCT, hematopoietic stem cell transplantation; MB, medulloblastoma; NA, not available; ORR, overall response rate; PD, progressive disease; PNET, primitive neuroectodermal tumor; PR, partial response; SD, stable disease; TMZ, temozolomide; VCR, vincristine; VIT, Vincristine + Temozolomide + Irinotecan.

a In these series there were patients with medulloblastoma who were not evaluable for response. Therefore, the number of responses is not equal to the number of patients with medulloblastoma included in the study.

b ORR/DCR was calculated as the proportion of evaluable patients for which response was available in each category (CR, PR, SD, and PD).

c Median ORR/DCR was calculated only based on the studies for which data on response (CR, PR, and SD) were available. It is expressed in percentage.

d Medulloblastoma/PNET cohort that could not be split with the data obtained from the report.

**Table 5:** Response rates of phase II studies including patients with medulloblastoma

	*N* (MB patients)	CR	PR	SD	PD	Objective Response Rate (%)	Disease control rate (%)	Reference (Year of publication)
Conventional chemotherapeutic single agent								
Oral methotrexate	18	0	0	6	11^a^	0	35	79 (2000)
Placitaxel	16	1	0	5	8^a^	7	43	80 (2001)
Idarubicin	21	0	1	6	11^a^	6	39	81 (2003)
Oxaliplatin	30	0	2	5	23	7	23	82 (2006)
Temozolomide	29^f^	1	3	7	14^a^	16	56	83 (2007)
Temozolomide	37	6	9	10	12	41	67	84 (2014)
Topotecan	2	0	0	0	2	0	0	85 (2006)
Docetaxel	20	0	1	18	18^b^	5	NA	86 (2006)
Irinotecan	25^f^	0	4	NA	NA	16	NA	87 (2007)
Rebeccamycin analog	7	0	0	0	7	0	0	88 (2008)
Vinorelbine	2	0	1	0	1	50	50	89 (2009)
Pemetrexed	10	0	0	1	9	0	11	23 (2013)
Total	217	8	21	58	116			
ORR/DCR^d^	–	ORR 29/207 = 14%		NA^c^	–	–	–	
Median objective response/disease control rate (Range)^e^					7 (0–50)		37 (0–67)	
Conventional chemotherapeutics combination								
Temozolomide + Irinotecan	66	1	20	26	15^a^	34	75	24 (2013)
Lobradimil + Carboplatin	6^f^	0	0	0	6	0	0	90 (2006)
Gemcitabine + Oxaliplatin	14	0	1	6	7	7	50	91 (2011)
Vinorelbine + CPM	7	0	0	1	6	0	14	92 (2012)
Total	93	1	21	33	34			
ORR/DCR	–	ORR 21/89 = 23%		DCR 53/89 = 59%	–	–	–	
Median objective response/disease control rate (Range)^e^					3.5 (0–34)		32 (0–75)	
Targeted agent monotherapy								
Tipifarnib	12	0	0	0	12	0	0	93 (2007)
Imatinib	8^f^	0	0	1	7	0	13	94 (2009)
Lapatinib	12	0	0	3	9	0	25	22 (2013)
Vismodegib	12	0	1	0	11	8	8	98 (2015)
Total	44	0	1	4	39			
ORR/DCR	–	ORR 1/44 = 2%		DCR 5/44 = 11%	–	–	–	
Median objective response/disease control rate (Range)^e^					0 (0–8)		11 (0–25)	
Targeted agent combination (*n* = 0)								
Chemotherapeutics + targeted agent in combination								
Bevacizumab + Irinotecan	10	NA	NA	NA	NA	NA	NA	25 (2013)
Multiagent metronomic	6	1	0	2	3	17	50	95 (2014)
Total	16	1	0	2	–	–	–	
ORR/DCR	–	ORR 1/6 = 17%		DCR 3/6 = 50%	–	–	–	
Median objective response/disease control rate (Range)^e^						17	50	
Chemotherapeutics + HSCT								
Multiagent conditioning	9	NA	NA	NA	NA	NA	NA	96 (2010)
CPM + Melphalan	22	NA	NA	NA	NA	NA	NA	97 (2008)
Total	31	–	–	–	–	–	–	
ORR/DCR	–	–	–	–	–	–	–	
Median objective response/disease control rate (Range)^e^						NA	NA	

CPM, cyclophosphamide; CR, complete response; DCR, disease control rate; HSCT, hematopoietic stem cell transplantation; MB, medulloblastoma; NA, not available; ORR, overall response rate; PD, progressive disease; PNET, primitive neuroectodermal tumor; PR, partial response; SD, stable disease.

a In these series there were patients with medulloblastoma who experienced early death or for whom disease evaluation was unknown. Therefore, the number of responses is not equal to the number of patients with medulloblastoma included in the study.

b In these series, 18 patients experienced either SD or PD but figures were presented together in the original manuscript and therefore could not be split in this table. One of the 20 patients was not evaluable.

c Calculation of DCR cannot be made because there were two studies for which data about SD and PD could not be obtained.

d ORR/DCR was calculated as the proportion of evaluable patients for whom response was available.

e Median ORR/DCR was calculated only based on the studies for which data on response (CR, PR, and SD) were available. It is expressed in percentage.

f Medulloblastoma/PNET cohort that could not be split with the data obtained from the report.

Conventional single‐agent chemotherapeutics yielded the highest response rates in phase I (median DCR 16%, 0–100) and II studies (median DCR 37%, 0–67). Within phase II trials there were three studies in which patients died of documented progressive disease before their first scheduled evaluation (*n* = 4 patients, 0.6% of 662 patients) 79, 80, 81.

### Response and outcome in medulloblastoma‐/PNET‐specific trials

Four studies were addressed exclusively to patients with medulloblastoma evaluating the smoothened (SMO) inhibitor vismodegib (*n* = 2) 26, 98, temozolomide, and etoposide 40, and the combination of temozolomide with irinotecan 24. In the phase II study evaluating temozolomide and irinotecan, ORR and DCR were 33% and 73%, respectively; 46.2% of the patients were progression free at 6 months and 79.7% were still alive, which is the best response obtained among these four studies, although with a short follow‐up for progression free 24. One study including patients with medulloblastoma and PNET, investigated temozolomide as a single agent 84. Within 37 patients with medulloblastoma, ORR was 46%, including six CR and a progression‐free survival rate among those with objective response at 6 and 12 months of 70.6% and 17.5%, respectively.

### Description of response and outcome by therapeutic class of agents

In this section we describe the results for specific therapeutic class of agents that have been tested more frequently.

### Platinum salts

Platinum salts were the most frequent class of agent tested (*n* = 15, 19%). Median ORR varied from 0 to 7% 37, 82 when used as a single agent, and up to 33% 47 when combined with etoposide and 100% 46 with irinotecan.

### Temozolomide

Temozolomide was the second most common agent tested (*n* = 13, 17%). Temozolomide containing studies have shown a median ORR of 16.5% (range, 0–100%) and a median DCR of 36.5% (range, 0–100%). Phase II studies containing temozolomide had a median ORR of 33% (range, 16–46) and a median DCR of 57% (range, 40–73). Toxicity is mainly represented by hematological and gastrointestinal events.

### Targeted therapies

Three different categories of targeted agents (*n* = 36) have been evaluated: small molecules (*n* = 30, 83%), antibodies (*n* = 5, 14%), and immunotherapeutic agents (*n* = 1, 3%).

### The smoothened (SMO) inhibitors

Three studies have evaluated two different SMO inhibitors. Sonidegib was evaluated in a phase I–II study where the cohort included patients with relapsed tumors potentially dependent on sonic hedgehog (Shh) signaling 27; 33 patients were included, 24 of whom had a medulloblastoma. ORR for the whole population was 6% (two CR in Hh‐activated medulloblastoma; of note, only 14 patients with medulloblastoma were evaluated with the 5‐gene Hh signature assay, and only the two patients who responded had an Hh‐activated medulloblastoma). In the phase I study of Vismodegib, seven of 33 patients were found to have Hh‐activated disease, of which only one responded (unsustained 8‐week CR, ORR 3%) 26. In the phase II part of the study, 12 other patients were included and only one experienced sustained response 98.

### Antiangiogenic therapies

A total of nine studies evaluated antiangioenic therapies. A phase II trial with multiagent oral antiangiogenic regimen in patients with medulloblastoma (*n* = 6) reported one CR (ORR 17%) and two disease stabilizations (DCR 50%) with a tolerable toxicity profile 95. The combination of bevacizumab with vincristine, irinotecan, and temozolomide resulted in one partial response after four cycles (3 months) allowing the patient to be consolidated with radiotherapy (ORR 100%) 73. The combination of bevacizumab and temsirolimus resulted in a 5‐month sustained disease stabilization in one of two patients included (DCR 50%) 68 and one patient receiving bevacizumab and irinotecan achieved a 14‐month disease stabilization (DCR 50%) 74. Other evaluated antiangiogenic agents such as cilengitide 52 or thalidomide and its analogs, either in monotherapy 56 or in combination with platinum agents 71, have yielded only short‐lasting disease stabilizations.

### Current and forthcoming molecularly stratified studies and targeted and immunotherapeutic agents in clinical trials for medulloblastoma patients

Fifty‐one studies were identified in the https://clinicaltrials.gov website, of which 20 were molecularly stratified studies and targeted/immunotherapeutic trials addressed to patients with medulloblastoma: five (25%) in first line and fifteen (75%) in second or subsequent lines (Table 6).

**Table 6:** Active and forthcoming molecularly stratified and tumor‐specific studies and targeted agents tested in clinical trials for medulloblastoma patients

First line treatments					
Population	Intervention	Phase	Sponsor	Responsible party	Reference
Classical MB WNT positive tumors and absence of other high‐risk clinical and molecular features^a^	Surgery + combination chemotherapy No radiotherapy	II	Academia	Sidney Kimmel Cancer Center	NCT02212574
Classical MB WNT positive tumors and absence of other high‐risk clinical and molecular features^a^	Surgery + Combination chemotherapy and reduced local and craniospinal irradiation	II	Academia	Children′s Oncology Group	NCT02724579
Low‐risk (LR)^b^ and standard‐risk (SR) MB patients	LR: Surgery + Radiotherapy and reduced radiotherapy and maintenance chemotherapy SR: Surgery + Radiotherapy (± carboplatin) and radiotherapy and maintenance chemotherapy	II–III	Academia	Universitätsklinikum Hamburg‐Eppendorf	NCT02066220 (PNET‐5)
WNT, SHH, and Non‐WNT or Non‐SHH MB patients	LR WNT tumors: Lower dose of radiotherapy and chemotherapy SHH patients: Value of adding vismodegib IR and HR Non‐WNT/Non‐SHH: Value of adding pemetrexed and gemcitabine	II	Academia	St. Jude Children′s Research Hospital	NCT01878617
Standard‐Risk MB patients	Postoperative radioimmunotherapy (intrathecal 131‐I‐3F8) Reduced doses of CSI, primary site boost, and standard adjuvant chemotherapy	II	Academia	Memorial Sloan Kettering Cancer Center	NCT00058370

Second and subsequent lines of treatment					
Population	Intervention	Phase	Sponsor		Reference
Studies with a specific cohort for medulloblastoma patients					
MB and PNET	Vaccine immunotherapy (TTRNA‐xALT)	I	Academia	University of Florida	NCT01326104
MB and ATRT	Modified measles virus (MV‐NIS)	I	Academia	University of California, San Francisco	NCT02962167
MB and CNS and other solid tumors	AZD1175 (Wee1 inhibitor) + Irinotecan	I	Academia	NCI	NCT02095132
MB and CNS tumors	Indoximod (IDO checkpoint inhibitor) + TMZ	I	Industry	NewLink Genetics Corporation	NCT02502708
MB	Metronomic and targeted antiangiogenesis therapy	II	Academia	Medical University of Vienna	NCT01356290
MB and other solid tumors (carcinoid, neuroblastoma and neuroendocrine tumors)	Dosimetry‐Guided 90Y‐DOTA‐tyr3‐Octreotide Peptide Receptor Radiotherapy	II	Academia	University of Iowa	NCT02441088
MB	TB‐403 (monoclonal antibody against placental growth factor [PlGF])	I–II	Industry	Oncurious NV	NCT02748135
Studies addressed to patients with relapsed malignancies including also medulloblastoma patients					
CNS tumors	Wild‐Type Reovirus in Combination With Sargramostim	I	Academia	Mayo Clinic	NCT02444546
CNS tumors	Palbociclib (CDK 4–6 inhibitor)	I	Academia	Pediatric Brain Tumor Consortium	NCT02255461
Solid tumors that are 8H9 reactive	Iodine I 131 monoclonal antibody 8H9	I	Academia	Memorial Sloan Kettering Cancer Center	NCT00089245
Solid tumors undergoing autologous hematopoietic stem cell transplantation	Antiangiogenic therapy: Cyclophosphamide or thalidomide beginning Day +30 (30 days posttransplant) and continued until at least Day +86	I	Academia	Washington University School of Medicine	NCT01661400
Solid tumors	Talazoparib (PARP inhibitor) + Irinotecan ± temozolomide	I	Academia	St. Jude Children′s Research Hospital	NCT02392793
Solid tumors and hematologic malignancies	Multiarm targeted therapies ± conventional chemotherapeutics (ESMART)	I–II	Academia	Gustave Roussy	NCT02813135
Solid tumors and hematologic malignancies	Multiarm targeted therapies (Pediatric MATCH)	II	Academia	National Cancer Institute	NCT03233204 NCT03213665 NCT03213678 NCT03213704 NCT03210714 NCT03155620
Solid tumors	Erlotinib (EGFR inhibitor) and TMZ	II	Academia	Washington University School of Medicine	NCT02689336

ATRT, atypical teratoid rhaboid tumor; CNS, central nervous system; CSI, craniospinal irradiation; HR, high risk; IR, intermediate risk; LR, low risk; MB, medulloblastoma; NCI, National Cancer Institute; PNET, primitive neuroectodermal tumors; PlGF, placental growth factor; SHH, sonic hedgehog; SR, standard risk; TMZ, temozolomide.

a High‐risk features are defined as metastatic disease, >1.5 cm^2^ postoperative residual tumor, presence of MYC or MYCN amplification, absence of nuclear beta‐catenin reactivity, and unfavorable histology (large‐cell or anaplastic subtypes).

b In the PNET V study the Low‐Risk group is defined as the WNT subgroup positivity.

## Discussion

The outcome of patients with medulloblastoma has improved over the last decades. This has been largely achieved as a result of international collaborative efforts through clinical trials 99. Still, outcome for those with metastatic disease, adverse molecular or cytogenetic features, infants 99, and relapsed or refractory patients 11 remains challenging.

In addition, for those who survive long‐term side effects are of major importance. Hearing and cognitive impairment can hamper independent living and these patients are endured an increased risk of stroke and secondary neoplasms 100, 101, 102, among other late effects.

Therefore, clinical trials are clearly needed to find new strategies to improve their outcome and reduce long‐term sequelae.

This study covers an expanded period of time in which new agents and strategies have been tested giving a precise landscape of the attempts to improve the outcome of patients with relapsed medulloblastoma.

Some limitations must be pointed out. Firstly, the search strategy was limited to articles indexed in Pubmed, those with results in https://clinicaltrials.gov, and references from selected studies. We did not search meetings’ abstracts books, where preliminary results from ongoing trials are presented before definitive publication. Secondly, results disclosing response need to be interpreted cautiously due to heterogeneity between studies as regards to eligibility criteria, patient population (e.g., first or subsequent relapse), and, more importantly, the limited number of patients with medulloblastoma in each trial. In addition, the radiological response criteria used across phase II studies were heterogeneous, with 75% using WHO and 21% using RECIST. Finally, we identified in phase II studies that true response rates to declare a drug active were heterogeneous, even when evaluating the same drug in similar scenarios. This means that a trial might be deemed successful or not based on how we predefine the true response rates. Activity data from historical controls are used to calculate true response rates for interventional clinical trials, although it still has major limitations 103. Yet randomized trials remain the best method to discern true effects in interventional studies.

Of note, only a small number of patients died of rapid disease progression before the first scheduled trial evaluation (4/662; 0.6) 79, 80, 81 and it has been shown that poor performance status at enrolment correlates with worse survival in children with brain tumors participating in phase I trials 104.

Objective response rates remain modest. Median ORR rate for patients with medulloblastoma was 0% (range, 0–100) in phase I studies and 6.5% (range, 0–50) in phase II. Median DCR for patients with medulloblastoma was 16% (0–100) in phase I studies and 25% (0–75) in phase II.

Among conventional chemotherapeutics, temozolomide‐containing regimens have shown most promising activity. Two studies, one in monotherapy 84 and another in combination with irinotecan 24, have shown the best results in a relatively large population, although follow up for disease‐free survival is short. Its tolerable toxicity profile and synergies with other chemotherapeutics and targeted agents make it an attractive compound to serve as backbone for new strategies. Indeed, temozolomide has been brought to frontline trials as maintenance therapy after intensive chemotherapy and hematopoietic stem cell transplantation in metastatic CNS‐PNET patients (NCT00936156).

The advent of the molecular classification of medulloblastoma in 2012 17 and the progressive implementation of molecular techniques able to clarify key biology aspects have permitted to improve our understanding of this disease and develop more specific strategies.

More recently, the identification of novel molecular subgroups has permitted to further stratify patients into four prognostic categories (favorable, standard, high, and very high risk) 105; this implies that our current frontline therapeutic approach needs to be revised.

In this sense, serial characterization of medulloblastomas at diagnosis and at the time of relapse has shown that medulloblastoma does not change subgroup at recurrence but have drastically different genomes than the primary disease, and that the pattern of recurrence is driven by subgroup affiliation rather than treatment 106 (e.g., SHH tumors recur mostly locally and groups 3 and 4 recur almost exclusively with metastases with prolonged long‐term postrecurrence survival). Future strategies addressed to patients with groups 3 and 4 medulloblastoma should consider intensification of treatments aimed at the metastatic compartment (e.g., intrathecal consolidation) 106.

Based on the fact that pediatric tumors evolve under therapy with emerging new molecular alterations 107 and behave differently at the time of relapse 106 or develop secondary events that require a complete distinct approach 106, several platforms in Europe (iTHER, INFORM) look to identify changes in the tumor molecular profile by comparing tissue from diagnosis with that at relapse in order to identify new therapeutic opportunities.

The sonic hedgehog pathway plays a critical role in normal cerebellar development; desmoplastic, nodular, and extensive nodularity subtypes are universally associated with Shh pathway activation. Alterations in this pathway are characteristics of one of the four molecular subgroups in medulloblastoma, the so‐called Shh group 2. The application of the first smoothened inhibitor showed extraordinary (although short‐lasting) response in first‐in‐human studies 108. But subsequent studies in selected Shh‐activated patients have yielded only limited and short‐lasting responses 26, 98. Nonetheless, prolonged complete responses have also been reported 27. For this reason, vismodegib is currently being evaluated as maintenance treatment postradiotherapy and chemotherapy for skeletally mature children with newly diagnosed standard‐risk Shh medulloblastoma (NCT01878617). Whether SMO inhibitors are called to play a major role in this subset of patients remains unclear. The genomic aberration relative to SMO is predictive of SMO inhibitor activity 98 and current efforts are focusing on identifying which subset of Hh‐activated tumors are more likely to respond by means of a complete molecular profiling. The Shh pathway can also be targeted at different levels to disrupt tumorigenesis and to overcome the limitations of single‐agent therapies; for instance, blocking GLI1 with arsenic trioxide 2, or combining SMO inhibitors with PI3K inhibitors 98, whose aberrations are frequent in this subset of patients.

Non‐WNT/Non‐SHH medulloblastomas comprise groups 3 and 4 of the molecular classification. Altogether they represent up to 60% of all medulloblastoma, but the underlying molecular drivers yet remain to be fully characterized and therefore no specific targeted treatments are available at present 2. A phase II clinical trial (NCT01878617) is currently evaluating the addition of pemetrexed and gemcitabine in consolidation. Both pemetrexed 23, 36 and gemcitabine 91 have been previously tested per separate in medulloblastoma patients. In our analysis, only the combination of gemcitabine with oxaliplatin was found to have promising results (one PR and six disease stabilizations of 14 treated medulloblastoma patients; ORR 7% and DCR 50%) 91. Interestingly, a recent preclinical study identified the combination of these two drugs as active, both in cellular assays and in mouse models of group 3 medulloblastoma 109, further supporting the interest of combination in prospective studies (NCT01878617). For patients with group 4 medulloblastomas, there may be a role for epigenetic‐based therapies, such as demethylating agents and histone deacetylase inhibitors 2, 99. The combination of vorinostat and retinoic acid resulted in a 5‐month disease stabilization 67, while no responses were seen when combining vorinostat with temozolomide 69 or with bortezomib 77.

Ongoing and forthcoming phase I‐II trials in medulloblastoma are addressed to specific cancer vulnerabilities (Table 6). New strategies look to identify genetic aberrations through exhaustive molecular screening, which permits patients with individual alterations to receive a coupled treatment (ESMART trial; NCT02813135).

In conclusion, this systematic review shows that there have been a large number of studies evaluating new therapies in children with medulloblastoma but with limited impact in their survival outcomes. The heterogeneity between trials in terms of their design and study population limits the generalization of those results and no randomized studies have been conducted. Temozolomide‐containing regimens are tolerable and have demonstrated antitumor activity against relapsed/refractory medulloblastoma. Future studies may consider using this drug as a backbone for new combinations. Targeted therapies have shown modest antitumor activity; SMO inhibitors are promising agents in Hh‐activated tumors, although still we need to identify which subset of patients can benefit more from this approach. New high‐throughput molecular platforms permitting to dissect and compare tumor biology at diagnosis and at relapse will allow identifying patients harboring specific genetic aberrations who are suitable candidates for new targeted therapies and therefore more likely to derive benefit from these novel agents.

## Conflict of Interest

The authors declare that they have no conflict of interest.

## Supporting information


**Data S1.** Search strategy (PUBMED).
