# Supplementary material for: Medulloblastoma in children and adolescents: a systematic review of contemporary phase I and II clinical trials and biology update
Source: Cancer Med. 2017 Oct 4;6(11):2606–24. doi: 10.1002/cam4.1171 (PMC5673921; doi:10.1002/cam4.1171)
Supplement: Supplementary file 1 — Data S1. Search strategy (PUBMED). [file CAM4-6-2606-s001.docx]

**Data S1. Search strategy (PUBMED):**

**- Medulloblatoma trials** (phase[All Fields] AND 1[All Fields] AND ("clinical trials as topic"[MeSH Terms] OR ("clinical"[All Fields] AND "trials"[All Fields] AND "topic"[All Fields]) OR "clinical trials as topic"[All Fields] OR "trial"[All Fields])) OR (phase[All Fields] AND 2[All Fields] AND ("clinical trials as topic"[MeSH Terms] OR ("clinical"[All Fields] AND "trials"[All Fields] AND "topic"[All Fields]) OR "clinical trials as topic"[All Fields] OR "trial"[All Fields])) AND ("medulloblastoma"[MeSH Terms] OR "medulloblastoma"[All Fields]).

- **Brain tumor trials** (phase[All Fields] AND 1[All Fields] AND ("clinical trials as topic"[MeSH Terms] OR ("clinical"[All Fields] AND "trials"[All Fields] AND "topic"[All Fields]) OR "clinical trials as topic"[All Fields] OR "trial"[All Fields])) OR (phase[All Fields] AND 2[All Fields] AND ("clinical trials as topic"[MeSH Terms] OR ("clinical"[All Fields] AND "trials"[All Fields] AND "topic"[All Fields]) OR "clinical trials as topic"[All Fields] OR "trial"[All Fields])) AND ("brain tumours"[All Fields] OR "brain neoplasms"[MeSH Terms] OR ("brain"[All Fields] AND "neoplasms"[All Fields]) OR "brain neoplasms"[All Fields] OR ("brain"[All Fields] AND "tumors"[All Fields]) OR "brain tumors"[All Fields]).

- **Solid tumor trials** (phase[All Fields] AND 1[All Fields] AND ("clinical trials as topic"[MeSH Terms] OR ("clinical"[All Fields] AND "trials"[All Fields] AND "topic"[All Fields]) OR "clinical trials as topic"[All Fields] OR "trial"[All Fields])) OR (phase[All Fields] AND 2[All Fields] AND ("clinical trials as topic"[MeSH Terms] OR ("clinical"[All Fields] AND "trials"[All Fields] AND "topic"[All Fields]) OR "clinical trials as topic"[All Fields] OR "trial"[All Fields])) AND (solid[All Fields] AND ("tumours"[All Fields] OR "neoplasms"[MeSH Terms] OR "neoplasms"[All Fields] OR "tumors"[All Fields])).
